# Differential patterns of lysosomal dysfunction are seen in the clinicopathological forms of primary progressive aphasia

**DOI:** 10.1007/s00415-023-12063-9

**Published:** 2023-11-02

**Authors:** Imogen J. Swift, Simon Sjödin, Johan Gobom, Ann Brinkmalm, Kaj Blennow, Henrik Zetterberg, Jonathan D. Rohrer, Aitana Sogorb-Esteve

**Affiliations:** 1 UK Dementia Research Institute at University College London, UCL Queen Square Institute of Neurology, University College London, London, UK; 2 Dementia Research Centre, Department of Neurodegenerative Disease, UCL Queen Square Institute of Neurology, University College London, Gower Street, London, WC1E 6BT UK; 3 Department of Psychiatry and Neurochemistry, Institute of Neuroscience and Physiology, The Sahlgrenska Academy at the University of Gothenburg, Mölndal, Sweden; 4 Clinical Neurochemistry Laboratory, Sahlgrenska University Hospital, Mölndal, Sweden; 5 Hong Kong Center for Neurodegenerative Diseases, Hong Kong, China

**Keywords:** Frontotemporal dementia, Primary progressive aphasia, Endo-lysosomal proteins, Ubiquitin

## Abstract

Increasing evidence implicates endo-lysosomal dysfunction in frontotemporal dementia (FTD). 18 proteins were quantified using a mass spectrometry assay panel in the cerebrospinal fluid of 36 people with the language variant of FTD, primary progressive aphasia (PPA) (including 13 with non-fluent variant (nfvPPA), 11 with semantic variant (svPPA), and 12 with logopenic variant (lvPPA)) and 19 healthy controls. The concentrations of the cathepsins (B, D, F, L1, and Z) as well as AP-2 complex subunit beta, ganglioside GM2 activator, beta-hexosaminidase subunit beta, tissue alpha l-fucosidase, and ubiquitin were decreased in nfvPPA compared with controls. In contrast, the concentrations of amyloid beta A4 protein, cathepsin Z, and dipeptidyl peptidase 2 were decreased in svPPA compared with controls. No proteins were abnormal in lvPPA. These results indicate a differential alteration of lysosomal proteins in the PPA variants, suggesting those with non-Alzheimer’s pathologies are more likely to show abnormal lysosomal function.

## Introduction

The primary progressive aphasias (PPA) are disorders characterized by focal degeneration of the brain regions involved in language function and can be divided into three main subtypes: the non-fluent or agrammatic variant (nfvPPA), the semantic variant (svPPA), and the logopenic variant (lvPPA) [1]. These three variants are distinguished by the type of linguistic deficits with which they present, as well as their neuroanatomical signatures and underlying pathology [2]. For example, nfvPPA is most commonly a primary tauopathy as seen in progressive supranuclear palsy or corticobasal degeneration [3, 4] and svPPA is in most cases a TDP-43 proteinopathy [5], both reflecting frontotemporal lobar degeneration (FTLD) pathology, whereas lvPPA is usually caused by Alzheimer’s disease (AD) pathology [6].

Knowledge about the underlying pathophysiology of the PPA disorders, which are all usually sporadic, is limited. However, in other forms of frontotemporal dementia (FTD), one of the pathways that has been highlighted in recent years as likely to be affected, is the endo-lysosomal system [7–9]. There is increasing evidence that suggests alterations in this system, leading to dysfunctional proteostasis, play a key role in neurodegenerative disorders. The role of lysosomes in the cell is to break down proteins and maintain homeostasis of the cell through processes like endocytosis and autophagy. Impairment of the normal function of the lysosomal pathway leads to protein accumulation and aggregation and, therefore, neurodegeneration. Lysosomal dysfunction has been implicated particularly in the pathophysiology of progranulin-related FTD, where progranulin itself and related lysosomal proteins such as prosaposin, the cathepsins, and glucocerebrosidase are affected [9]. However, this has yet to be investigated in sporadic PPA. The ubiquitin–proteasome system (UPS) is similarly a major intracellular protein degradation system whose dysfunction has been associated with many neurological diseases including AD and amyotrophic lateral sclerosis (ALS) [10]. Disruption of the UPS leads to deficits in the clearance of misfolded proteins, in turn causing intracellular protein aggregation, cytotoxicity, and cell death [11]. Furthermore, it has been demonstrated that the UPS plays a key role degrading TDP-43 [12], and clearing hyperphosphorylated tau, as well as degrading intra-neuronal insoluble tau aggregates [13]. Overall, the endo-lysosomal network, therefore, plays an important role in the clearance of the core proteins that aggregate within FTD spectrum disorders.

One way of measuring a change in cellular function in vivo is to develop fluid biomarkers to assess a change in the concentration of proteins in different parts of the pathway [14]. In this study, we measured the cerebrospinal fluid (CSF) concentration of a panel of proteins involved in both the endo-lysosomal and ubiquitin–protease system to investigate the presence of dysfunction in these pathways within the PPA disorders.

## Methods

### Participants

Thirty-six people with sporadic PPA and available CSF were recruited through the Longitudinal Investigation of FTD (LIFTD) study at University College London (Table 1): 13 nfvPPA, 11 svPPA, and 12 lvPPA, diagnosed according to current consensus criteria [2]. All cases were negative for any of the genes that are causative of FTD including the *C9orf72* expansion. Nineteen healthy controls were also recruited through the LIFTD study over the same time period. All patients with lvPPA had a biomarker profile consistent with underlying Alzheimer’s disease: mean (standard deviation) total tau/Aβ42 ratio of 3.2 (2.2) with a range of 1.2–8.3 where > 1 is considered abnormal. All nfvPPA and svPPA participants and all controls had a ratio of < 1.

**Table 1:** Demographics of the primary progressive aphasia groups and health controls. Values are shown as mean (standard deviation)

	Controls	svPPA	nfvPPA	lvPPA
Number of participants	19	11	13	12
Age at CSF collection	63.5 (6.9)	60.5 (5.9)	67.0 (6.3)	66.7 (6.3)
Sex (% male)	47.4	54.5	53.8	50
Disease duration at CSF collection (years)	N/A	4.6 (2.0)	4.5 (1.9)	3.6 (2.2)
Ab42 (pg/mL)	999.9 (235.4)	879.7 (259.5)	845.6 (318.3)	439.8 (159.4)
Total tau (pg/mL)	325.7 (93.3)	355.7 (152.9)	405.8 (184.7)	1206.0 (555.4)
Total tau/Ab42 ratio	0.3 (0.1)	0.4 (0.1)	0.5 (0.3)	3.2 (2.2)

### CSF samples

CSF was collected from all participants in polypropylene tubes through a lumbar puncture and centrifuged to remove insoluble material and cells. Supernatants were aliquoted and stored at − 80 °C within 2 h after withdrawal. CSF Aβ42, total tau, and phosphorylated tau concentrations were measured using commercially available enzyme-linked immunosorbent assays (INNOTEST; Fujirebio Europe, Ghent, Belgium), according to the manufacturer’s instructions.

### Sample digestion and solid-phase extraction

Digestion and solid-phase extraction (SPE) were performed as described previously [15, 16]. One hundred μL of CSF from subject samples was mixed with internal standard and then reduced and alkylated with 1,4-dithiothreitol and iodoacetamide, respectively. The samples were then digested using sequencing grade modified trypsin (Promega Co., Madison, WI, USA). Trypsination was ended by the addition of trifluoroacetic acid and was followed by SPE using Oasis HLB 96-well μElution Plates (2 mg sorbent and 30 μm particle size; Waters Co., Milford, MA, USA) according to the generic protocol of the manufacturer. As the final step, the samples were eluted in methanol and dried by vacuum centrifugation. The samples were frozen and stored at − 80 °C pending analysis.

### Parallel reaction monitoring-mass spectrometry

**Fig. 1:**
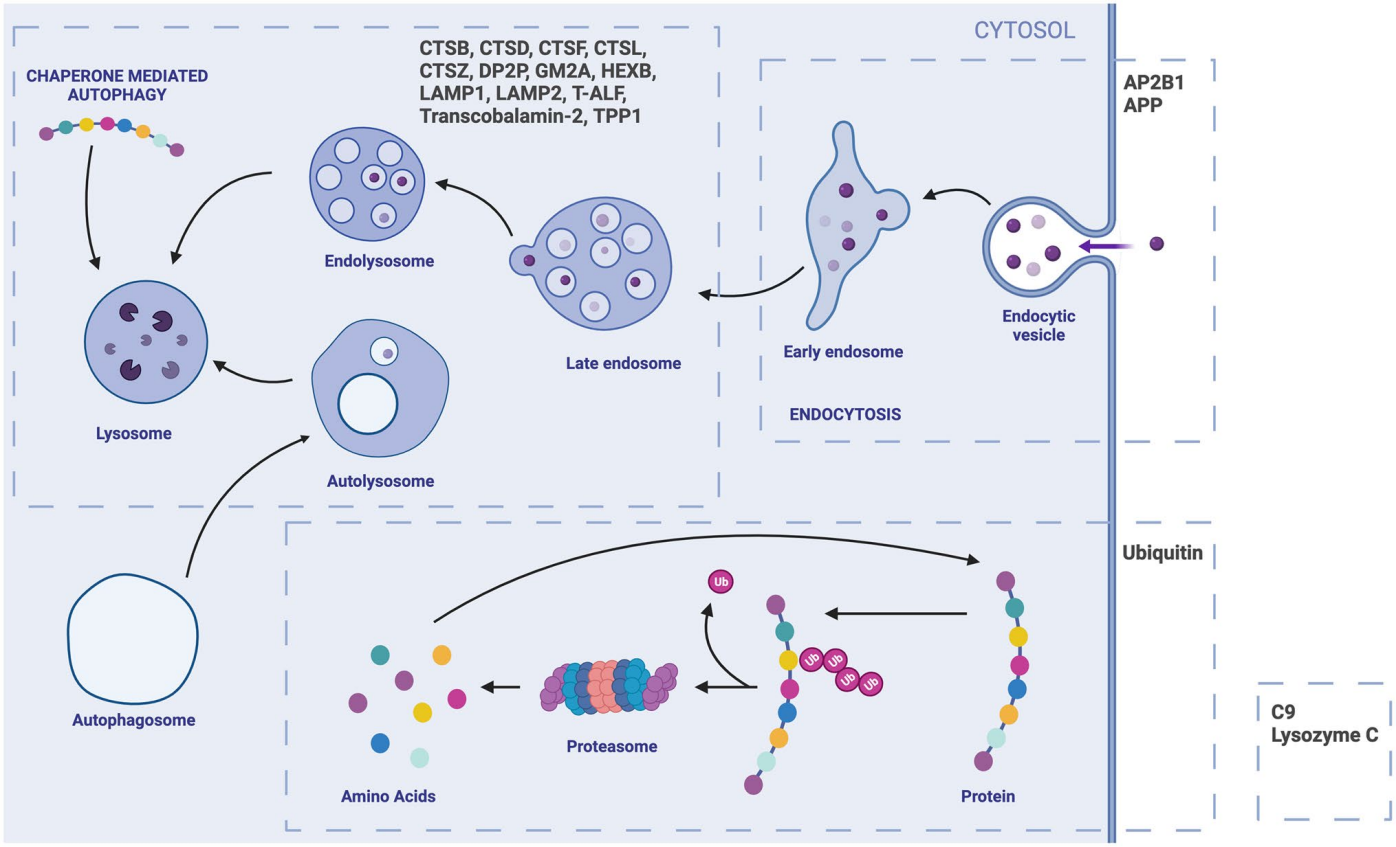
Schematic diagram showing the endo-lysosomal system and the proteins measured in the study (see main text for abbreviations)

Eighteen proteins (49 peptides) were quantified by parallel reaction monitoring-mass spectrometry (PRM-MS) and are shown in Fig. 1 as well as being listed in Table 2: AP-2 complex subunit beta (AP2B1), amyloid beta A4 protein (APP), complement component C9, cathepsins B, D, F, L1, Z, dipeptidyl peptidase 2 (DPP2) ganglioside GM2 activator (GM2A), beta-hexosaminidase subunit beta (HEXB), lysosome-associated membrane glycoprotein 1 (LAMP1), lysosome-associated membrane glycoprotein 2 (LAMP2), lysozyme C, tissue alpha-l-fucosidase T-ALF, transcobalamin-2, tripeptidyl peptidase 1 (TPP1), and ubiquitin. PRM-MS analysis was performed as described previously [16] using an UltiMate 3000 standard-LC system (Thermo Fisher Scientific Inc., Waltham, MA, USA) and a Hypersil GOLD HPLC C_18_ column (length 200 mm; inner diameter 2.1 mm; particle size 1.9 μm; Thermo Fisher Scientific Inc.), and a Q Exactive mass spectrometer (Thermo Fisher Scientific Inc.). Electrospray ionization was performed in positive ion mode with a Heated Electrospray Ionization (HESI-II) probe (Thermo Fisher Scientific Inc.). Acquisition of single microscans was performed in PRM mode with an isolation window of *m/z* 3, a resolution setting 70 k, an AGC target 1 × 10^6^, a maximum injection time 300 ms, and fragmentation with beam type collision-induced dissociation (HCD). Peak detection and area integration were performed using Skyline v3.6 [17], targeting [M + H]^1+^ y-ions with a data-independent acquisition method setting and a fixed isolation window of m/z 3 and an orbitrap analyzer resolution setting of 70 k at m/z 200. The concentration of the peptides is expressed as a ratio between the sum of fragment ions of the tryptic peptide against the corresponding heavy labeled internal standard peptide (L/H peptide ratio). For the proteins for which more than one peptide was quantified, the peptide with the best analytical performance (lowest coefficient of variation) was selected.

**Table 2:** List of proteins and their corresponding peptides included in the analysis

Protein name	UniProtKB accession	Abbreviation	Peptide	Controls	lvPPA	nfvPPA	svPPA
AP-2 complex subunit beta	P63010	AP2B1	712–719	0.90 (0.27)	1.15 (0.43)	0.68 (0.24)*	0.80 (0.40)
			835–842	1.10 (0.41)	1.46 (0.62)	0.81 (0.29)*	0.92 (0.48)
			868–878	1.58 (0.60)	1.93 (0.79)	1.21 (0.64)*	1.33 (0.72)
Amyloid beta A4 protein	P05067	APP	289–301	1.43 (0.49)	1.71 (0.67)	1.17 (0.55)*	1.02 (0.30)*
			439–450	1.75 (0.70)	2.52 (1.25)	1.35 (0.68)*	1.40 (0.65)*
Complement component C9	P02748	C9	146–154	1.05 (0.78)	1.25 (1.26)	1.17 (0.73)	**1.69 (0.95)**
			186–194	0.50 (0.24)	0.61 (0.46)	0.47 (0.20)*	0.61 (0.34)
			232–242	2.15 (1.010	2.80 (2.14)	2.05 (0.87)*	3.01 (2.04)
			473–483	2.37 (1.14)	2.88 (2.06)	2.12 (0.88)	3.40 (2.69)
			497–508	3.40 (1.66)	4.52 (3.40)	3.22 (1.37)	5.15 (4.23)
Cathepsin B	P07858	Cathepsin B	58–71	0.52 (0.18)	0.63 (0.23)	0.46 (0.12)*	0.51 (0.23)
			80–87	0.79 (0.28)	0.90 (0.25)	0.70 (0.19)*	0.69 (0.20)
			210–220	0.57 (0.17)	0.65 (0.18)	0.48 (0.11)*	0.48 (0.15)*
Cathepsin D	P07339	Cathepsin D	55–72	0.97 (0.49)	1.25 (0.58)	0.88 (0.29)*	0.84 (0.50)
			112–122	1.32 (0.39)	1.50 (0.58)	1.15 (0.24)*	1.31 (0.72)
			349–357	0.96 (0.27)	1.06 (0.30)	0.90 (0.18)	0.84 (0.15)
Cathepsin F	Q9UBX2	Cathepsin F	103–116	0.54 (0.14)	0.64 (0.25)	0.45 (0.12)*	0.55 (0.29)
			236–345	0.90 (0.22)	1.06 (0.32)	0.78 (0.20)*	0.80 (0.23)
			266–278	1.12 (0.33)	1.38 (0.63)	0.96 (0.26)*	1.16 (0.59)
			442–450	1.01 (0.27)	1.17 (0.42)	0.87 (0.25)*	0.96 (0.38)
Cathepsin L1	P07711	Cathepsin L1	105–116	1.16 (0.37)	1.34 (0.50	0.96 (0.27)*	1.08 (0.42)
Cathepsin Z	Q9UBR2	Cathepsin Z	39–47	1.15 (0.44)	1.28 (0.48)	1.07 (0.39)	0.92 (0.37)
			261–270	1.00 (0.32)	1.01 (0.24)	0.82 (0.21)*	0.75 (0.24)*
Dipeptidyl peptidase 2	Q9UHL4	DPP2	40–47	1.38 (0.86)	1.34 (0.79)	1.02 (0.30)	0.80 (0.35)*
			113–123	1.66 (0.98)	1.75 (0.84)	1.41 (0.45)	1.22 (0.60)
			449–462	0.84 (0.61)	0.87 (0.38)	0.64 (0.21)	0.50 (0.27)*
Ganglioside GM2 activator	P17900	GM2A	89–96	0.92 (0.18)	1.04 (0.39)	0.82 (0.28)	0.87 (0.27)
			170–179	1.38 (0.35)	1.58 (0.73)	1.15 (0.37)*	1.25 (0.47)
Beta-hexosaminidase subunit beta	P07686	HEXB	285–300	1.69 (0.61)	1.87 (0.77)	1.33 (0.42)*	1.59 (0.81)
			391–400	1.14 (0.38)	1.22 (0.51)	0.90 (0.26)	0.99 (0.48)*
Lysosome-associated membrane glycoprotein 1	P11279	LAMP1	138–146	1.28 (0.35)	1.46 (0.49)	1.14 (0.36)	1.06 (0.28)*
			327–337	1.28 (0.42)	1.66 (0.66)	1.27 (0.46)	1.17 (0.51)
			357–363	0.97 (0.33)	1.31 (0.58)	0.89 (0.33)*	0.91 (0.38)
Lysosome-associated membrane glycoprotein 2	P13473	LAMP2	133–144	3.38 (0.95)	3.66 (1.37)	2.96 (1.06)	3.18 (1.37)
			145–152	1.19 (0.33)	1.48 (0.62)	1.13 (0.43)	1.31 (0.71)
			153–161	0.06 (0.02)	0.06 (0.03)	0.05 (0.02)	0.05 (0.01)
Lysozyme C	P61626	Lysozyme C	52–59	0.80 (0.37)	0.90 (0.47)	0.63 (0.22)	0.75 (0.57)*
			69–80	0.45 (0.18)	0.51 (0.24)	0.36 (0.11)	0.51 (0.51)
Tissue alpha-l-fucosidase	P04066	T-ALF	114–130	0.11 (0.04)	0.12 (0.05)	0.09 (0.02)*	0.10 (0.05)
			163–173	1.27 (0.49)	1.45 (0.51)	1.05 (0.24)*	1.06 (0.46)
			344–354	1.34 (0.50)	1.54 (0.54)	1.11 (0.25)*	1.20 (0.57)
Transcobalamin-2	P20062	Transcobalamin-2	45–59	1.60 (0.56)	1.83 (0.70)	1.33 (0.46)*	1.48 (0.86)
			300–313	0.80 (0.29)	0.97 (0.39)	0.68 (0.27)*	0.80 (0.54)
			393–399	1.06 (0.39)	1.29 (0.41)	0.99 (0.31)	1.19 (0.76)
Tripeptidyl peptidase 1	O14773	TPP1	61–78	1.54 (0.56)	1.64 (0.67)	1.39 (0.45)	1.42 (0.62)
			246–259	1.34 (0.44)	1.45 (0.55)	1.11 (0.29)	1.77 (2.13)
			507–520	0.95 (0.35)	1.06 (0.52)	0.83 (0.25)	0.87 (0.41)
Ubiquitin	P0CG48	Ubiquitin	12–27	1.52 (0.40)	1.87 (0.59)	1.23 (0.42)*	1.35 (0.55)
			64–72	0.82 (0.21)	0.99 (0.31)	0.65 (0.22)*	0.78 (0.41)

Mean (standard deviation) of the concentration of each peptide for each group is indicated in the corresponding column. Concentrations are expressed as a ratio between measured area of the tryptic peptide against the corresponding internal standard heavy label peptide. Peptides significantly altered in the corresponding group when compared to the control group are shown in bold, while significant differences compared with lvPPA are shown by an asterisk

### Statistical analyses

All statistical analyses were performed in STATA (v.16) and RStudio (R version 4.0.2). The Shapiro–Wilk test was performed to determine the normality of distribution of each endo-lysosomal marker in each group. The levels of each endo-lysosomal and ubiquitin protein were compared between groups using a linear regression model adjusting for age at CSF sample collection and sex; bootstrapping with 2000 repetitions was used if the measures were not normally distributed.

## Results

**Fig. 2:**
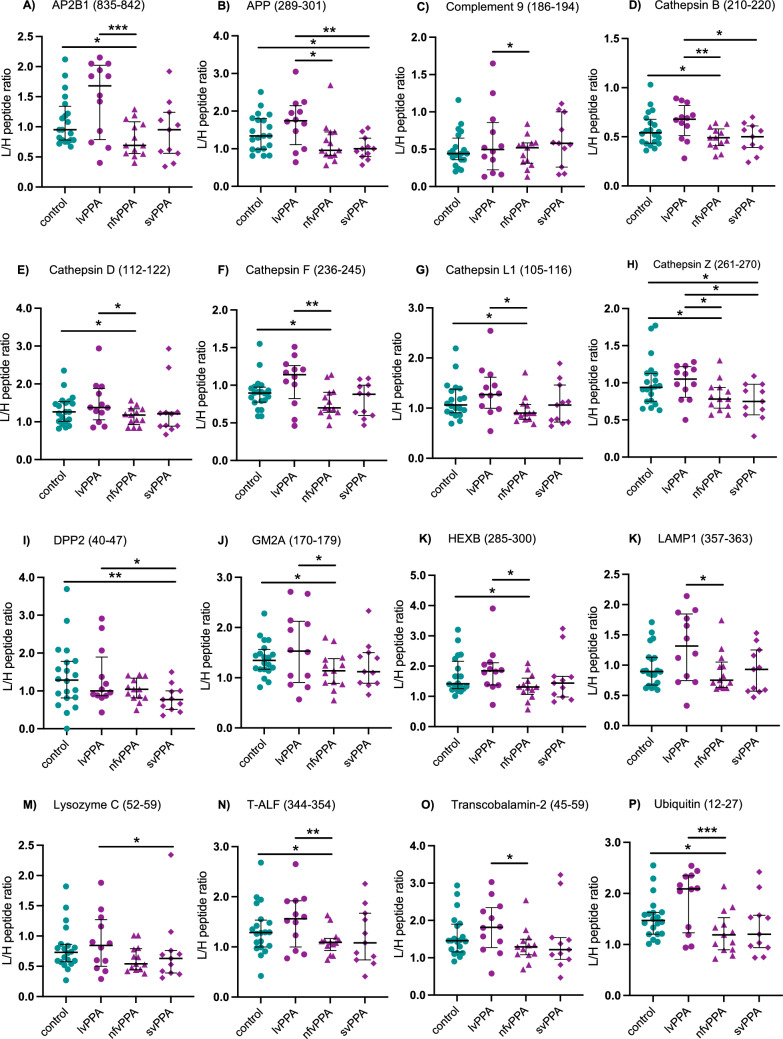
Altered CSF endo-lysosomal protein concentration in nfvPPA and svPPA groups compared to controls and lvPPA. Concentrations are expressed as a ratio between measured area of the tryptic peptide against the corresponding internal standard heavy label peptide (L/H peptide ratio). *p* values: *p ≤ 0.05, **p ≤ 0.01, ***p ≤ 0.001, and ****p ≤ 0.0001. The bars indicate the median and interquartile range. *AP2B1* AP-2 complex subunit beta, *APP* amyloid beta A4 protein, *DPP2* dipeptidyl peptidase 2, *GM2A* ganglioside GM2 activator, *HEXB* beta-hexosaminidase subunit beta, *LAMP1* lysosome-associated membrane glycoprotein 1, *T-ALF* tissue alpha-l-fucosidase

The concentration of ten out of the 18 proteins was lower in nfvPPA compared with both controls and lvPPA: AP2B1, cathepsins B, D, F, L1 and Z, GM2A, HEXB, T-ALF, and ubiquitin (Table 2, Fig. 2). Four further proteins were lower in nfvPPA compared with lvPPA: APP, complement 9, LAMP1, and transcobalamin-2 (Table 2, Fig. 2).

Three proteins were at lower levels in svPPA compared with both controls and lvPPA: APP, cathepsin Z, and DPP2 (Table 2, Fig. 2). Three additional proteins were lower in svPPA compared with lvPPA: cathepsin B, LAMP1, and lysozyme C (Table 2, Fig. 2). There were no significant differences between the lvPPA and controls, although for many of the peptides, there was a trend to higher levels in the lvPPA group.

Group differences are summarized in Table 3.

**Table 3:** Summary of the changes in each disease group

Change	Protein (significant peptides/peptides measured)
↓ nfvPPA vs control	AP2B1 (2/3); Cathepsin B (1/3); Cathepsin D (1/3); Cathepsin F (3/4); Cathepsin L1 (1/1); Cathepsin Z (1/2); GM2A (1/2); HEXB (1/2); T-ALF (3/3); Ubiquitin (2/2)
↓ nfvPPA vs lvPPA	AP2B1 (3/3); APP (2/2); Complement 9 (2/4); Cathepsin B (3/3); Cathepsin D (2/3); Cathepsin F (4/4); Cathepsin L1 (1/1); Cathepsin Z (1/2); GM2A (1/2); HEXB (1/2); LAMP1 (1/3); T-ALF (3/3); Transcobalamin-2 (2/3); Ubiquitin (2/2)
↓ svPPA vs control	APP (1/2); Cathepsin Z (1/2); DPP2 (2/3)
↑svPPA vs control	Complement 9 (1/4)
↓ svPPA vs lvPPA	APP (2/2); Cathepsin B (1/3); Cathepsin Z (1/2); DPP2 (2/3); HEXB (1/2); LAMP1 (1/3); Lysozyme C (1/2)

The number of peptides altered for each of the proteins out of the total number of peptides (i.e., peptides altered/total) is shown in parentheses

## Discussion

In this study, we show that there are abnormalities in the CSF concentrations of proteins associated with endocytosis, lysosomal function, and the ubiquitin–proteasome system in PPA. Interestingly, there was a decrease in the concentrations of proteins in those with a form of FTLD pathology (i.e., svPPA and nfvPPA) with an opposite trend in those with underlying AD pathology (lvPPA). The nfvPPA (usually due to a tauopathy) showed changes across multiple proteins, suggesting dysfunction in endocytosis and chaperone-mediated autophagy in this disorder. A more limited set of proteins were decreased in svPPA (usually a TDP-43 proteinopathy), but nonetheless, indicating abnormalities in the endo-lysosomal pathway in this condition.

The cathepsins are proteases whose key function in the lysosome is the degradation of proteins [18, 19]. To our knowledge, there are no previous reports on the levels of cathepsins in the biofluids of people with PPA, with the only previous study in an unspecified FTD cohort showing an increase in cathepsin D in plasma exosomes [7]. CSF cathepsins have been poorly studied in general in neurodegenerative diseases with few studies investigating their concentrations, e.g., a previous study reported a decrease in cathepsin B and cathepsin F in Parkinson’s disease (PD) when compared to controls and prodromal AD [16]; while in another study, cathepsin D was increased in AD [20]. In our study, while multiple cathepsins are decreased in nfvPPA (B, D, F, L1, and Z), only cathepsins B and Z are decreased in both nfvPPA and svPPA, particularly in relation to lvPPA. Interestingly, cathepsin D has been particularly implicated in progranulin-related FTD pathophysiology before [21] and clearly, further work is needed to understand how these other cathepsins are involved in these other pathological forms of FTD.

We further report a significant decrease in HEXB, a protein involved in chaperone-mediated autophagy, in nfvPPA in this study. To our knowledge, this has not been previously reported to be altered in the CSF of neurodegenerative disorders [16]. Lysosomal β-hexosaminidase A is a heterodimeric complex composed of HEXB and subunit alpha [22]. β-hexosaminidase A hydrolyses ganglioside GM2 with the aid of ganglioside GM2 activator [23], which of particular interest, is also decreased in the nfvPPA.

The LAMP proteins are also implicated in lysosomal autophagy, with functions in vesicle fusion [24], preserving lysosomal integrity and lysosomal exocytosis [25]. LAMP1 has been recently studied as a potential candidate marker of lysosomal alteration in neurodegenerative diseases with decreases shown in CSF in PD [8, 16, 26], although it has been previously reported to be unaltered in FTD [7]. Both proteins have been investigated in AD, with increases shown in previous studies [27]. However, in this study, there is a decrease in LAMP1 but not LAMP2, in the FTLD-related disorders, a key difference from lvPPA, a disorder with AD pathology.

Endocytosis and clathrin-mediated formation of the early endosome are the starting points of the endo-lysosomal pathway. AP2B1 and APP are implicated in this early stage of the lysosomal system [28–30]. AP2B is specifically altered in the nfvPPA group and has been reported to be significantly increased in AD and decreased in PD [16]. Our results regarding AP2B1 and APP suggest that the early stages of the lysosomal pathway are specifically altered in the FTLD-linked disorders (and potentially particularly the primary tauopathies) when compared to those disorders with underlying AD pathology.

Finally, we also studied the levels of ubiquitin in CSF. A key role of ubiquitin involves labeling proteins for degradation by the proteasome [31], but it also has a multitude of functions as a post-translational modification [32]. It has been reported to be increased in AD [16, 33]; however, our results in the FTLD-associated disorders parallel what has been found in other neurodegenerative diseases such as PD in which ubiquitin levels are decreased when compared to controls and AD [16].

Overall, the present study shows a decrease in protein degradation in FTLD-associated disorders nfvPPA and svPPA when compared to the AD group (lvPPA) and controls. We also see a trend to an increase in the levels of the proteins measured in the lvPPA group similar to that seen in typical amnestic AD. These results suggest a dysfunction of endo-lysosomal and ubiquitin systems in the FTD spectrum that will lead to a decrease in the degradation of proteins and possible accumulation.

There are a number of limitations of the study. We did not have access to detailed behavioral or neuropsychometry data within the cohort and it would be useful for future studies to investigate the correlation of clinical features with endo-lysosomal proteins and ubiquitin levels. The presence of co-morbidities such as systemic disease, mood disorders, and cerebrovascular disease (including for the latter, the presence of white matter hyperintensities on MRI) was also not evaluated: their effect on lysosomal-associated protein levels would be important to investigate in further analyses. While each group was of similar disease duration (time since symptom onset), participants were on average around 3–5 years into their illness. It would, therefore, be helpful to study both people very early in their clinical syndrome as well as to investigate longitudinal change in endo-lysosomal proteins and ubiquitin in PPA to understand the temporal relationship within the disease.

## Conclusions

This study highlights the complex endo-lysosomal system in the different variants of PPA and shows clear differences between those with AD and FTLD pathology. Our results establish a baseline for further study of the role of endo-lysosomal and ubiquitin proteins in PPA with the potential role of lysosomal dysfunction as a therapeutic target in these sporadic disorders, an important area of future research.

## Data Availability

The datasets used and/or analyzed during the current study are available from the corresponding author on reasonable request.

## References

[1] Marshall CR, Hardy CJD, Volkmer A, Russell LL, Bond RL, Fletcher PD, Clark CN, Mummery CJ, Schott JM, Rossor MN, Fox NC, Crutch SJ, Rohrer JD, Warren JD (2018). Primary progressive aphasia: a clinical approach. J Neurol.

[2] Gorno-Tempini ML, Hillis AE, Weintraub S, Kertesz A, Mendez M, Cappa SF, Ogar JM, Rohrer JD, Black S, Boeve BF, Manes F, Dronkers NF, Vandenberghe R, Rascovsky K, Patterson K, Miller BL, Knopman DS, Hodges JR, Mesulam MM, Grossman M (2011). Classification of primary progressive aphasia and its variants. Neurology.

[3] Graff-Radford J, Duffy JR, Strand EA, Josephs KA (2012). Parkinsonian motor features distinguish the agrammatic from logopenic variant of primary progressive aphasia. Parkinsonism Relat Disord.

[4] Kremen SA, Mendez MF, Tsai PH, Teng E (2011). Extrapyramidal signs in the primary progressive aphasias. Am J Alzheimers Dis Other Demen.

[5] Hodges JR, Patterson K (2007). Semantic dementia: a unique clinicopathological syndrome. Lancet Neurol.

[6] Henry ML, Gorno-Tempini ML (2010). The logopenic variant of primary progressive aphasia. Curr Opin Neurol.

[7] Goetzl EJ, Boxer A, Schwartz JB, Abner EL, Petersen RC, Miller BL, Kapogiannis D (2015). Altered lysosomal proteins in neural-derived plasma exosomes in preclinical Alzheimer disease. Neurology.

[8] Boman A, Svensson S, Boxer A, Rojas JC, Seeley WW, Karydas A, Miller B, Kagedal K, Svenningsson P (2016). Distinct lysosomal network protein profiles in Parkinsonian syndrome cerebrospinal fluid. J Parkinsons Dis.

[9] Parnetti L, Balducci C, Pierguidi L, De Carlo C, Peducci M, D’Amore C, Padiglioni C, Mastrocola S, Persichetti E, Paciotti S, Bellomo G, Tambasco N, Rossi A, Beccari T, Calabresi P (2009). Cerebrospinal fluid beta-glucocerebrosidase activity is reduced in Dementia with Lewy Bodies. Neurobiol Dis.

[10] Neumann M, Sampathu DM, Kwong LK, Truax AC, Micsenyi MC, Chou TT, Bruce J, Schuck T, Grossman M, Clark CM, McCluskey LF, Miller BL, Masliah E, Mackenzie IR, Feldman H, Feiden W, Kretzschmar HA, Trojanowski JQ, Lee VMY (2006). Ubiquitinated TDP-43 in frontotemporal lobar degeneration and amyotrophic lateral sclerosis. Science.

[11] Schwartz AL, Ciechanover A (2009). Targeting proteins for destruction by the ubiquitin system: implications for human pathobiology. Annu Rev Pharmacol Toxicol.

[12] Scotter EL, Vance C, Nishimura AL, Lee YB, Chen HJ, Urwin H, Sardone V, Mitchell JC, Rogelj B, Rubinsztein DC, Shaw CE (2014). Differential roles of the ubiquitin proteasome system and autophagy in the clearance of soluble and aggregated TDP-43 species. J Cell Sci.

[13] Jiang S, Bhaskar K (2020). Degradation and transmission of tau by autophagic-endolysosomal networks and potential therapeutic targets for tauopathy. Front Mol Neurosci.

[14] Blennow K, Zetterberg H, Fagan AM (2012). Fluid biomarkers in Alzheimer disease. Cold Spring Harb Perspect Med.

[15] Brinkmalm G, Sjödin S, Simonsen AH, Hasselbalch SG, Zetterberg H, Brinkmalm A, Blennow K (2018). A parallel reaction monitoring mass spectrometric method for analysis of potential CSF biomarkers for Alzheimer’s disease. Proteomics Clin Appl.

[16] Sjödin S, Brinkmalm G, Öhrfelt A, Parnetti L, Paciotti S, Hansson O, Hardy J, Blennow K, Zetterberg H, Brinkmalm A (2019). Endo-lysosomal proteins and ubiquitin CSF concentrations in Alzheimer’s and Parkinson’s disease. Alzheimers Res Ther.

[17] MacLean B, Tomazela DM, Shulman N, Chambers M, Finney GL, Frewen B, Kern R, Tabb DL, Liebler DC, MacCoss MJ (2010). Skyline: an open source document editor for creating and analyzing targeted proteomics experiments. Bioinformatics.

[18] Zaidi N, Maurer A, Nieke S, Kalbacher H (2008). Cathepsin D: a cellular roadmap. Biochem Biophys Res Commun.

[19] Turk V, Stoka V, Vasiljeva O, Renko M, Sun T, Turk B, Turk D (2012). Cysteine cathepsins: from structure, function and regulation to new frontiers. Biochim Biophys Acta.

[20] Schwagerl AL, Mohan PS, Cataldo AM, Vonsattel JP, Kowall NW, Nixon RA (1995). Elevated levels of the endosomal-lysosomal proteinase cathepsin D in cerebrospinal fluid in Alzheimer disease. J Neurochem.

[21] Valdez C, Wong YC, Schwake M, Bu G, Wszolek ZK, Krainc D (2017). Progranulin-mediated deficiency of cathepsin D results in FTD and NCL-like phenotypes in neurons derived from FTD patients. Hum Mol Genet.

[22] Lemieux MJ, Mark BL, Cherney MM, Withers SG, Mahuran DJ, James MNG (2006). Crystallographic structure of human beta-hexosaminidase A: interpretation of Tay-Sachs mutations and loss of GM2 ganglioside hydrolysis. J Mol Biol.

[23] Kolter T, Sandhoff K (2010). Lysosomal degradation of membrane lipids. FEBS Lett.

[24] Huynh KK, Eskelinen EL, Scott CC, Malevanets A, Saftig P, Grinstein S (2007). LAMP proteins are required for fusion of lysosomes with phagosomes. EMBO J.

[25] Saftig P, Klumperman J (2009). Lysosome biogenesis and lysosomal membrane proteins: trafficking meets function. Nat Rev Mol Cell Biol.

[26] Chu Y, Dodiya H, Aebischer P, Olanow CW, Kordower JH (2009). Alterations in lysosomal and proteasomal markers in Parkinson’s disease: relationship to alpha-synuclein inclusions. Neurobiol Dis.

[27] Armstrong A, Mattsson N, Appelqvist H, Janefjord C, Sandin L, Agholme L, Olsson B, Svensson S, Blennow K, Zetterberg H, Kågedal K (2014). Lysosomal network proteins as potential novel CSF biomarkers for Alzheimer’s disease. Neuromolecular Med.

[28] Haass C, Kaether C, Thinakaran G, Sisodia S (2012). Trafficking and proteolytic processing of APP. Cold Spring Harb Perspect Med.

[29] Müller UC, Zheng H (2012). Physiological functions of APP family proteins. Cold Spring Harb Perspect Med.

[30] McMahon HT, Boucrot E (2011). Molecular mechanism and physiological functions of clathrin-mediated endocytosis. Nat Rev Mol Cell Biol.

[31] Hershko A, Ciechanover A (1998). The ubiquitin system. Annu Rev Biochem.

[32] Komander D, Rape M (2012). The ubiquitin code. Annu Rev Biochem.

[33] Blennow kaj T, Davidsson P, Wallin A, Gottfries CG, Svennerholm L,  (1994). Ubiquitin in cerebrospinal fluid in Alzheimer’s disease and vascular dementia. Int Psychogeriatr.

